# Neuroprotective effects of Coenzyme Q10 in ischemia-reperfusion injury via inflammation and oxidative stress reduction in adult male rats

**DOI:** 10.25122/jml-2023-0099

**Published:** 2023-10

**Authors:** Zainab Ali Fakharaldeen, Ahmed Al-Mudhafar, Sarmad Nory Gany, Ali Nisoom Radhi, Najah Rayish Hadi

**Affiliations:** 1 Department of Pharmacology and Therapeutics, Faculty of Medicine, University of Kufa, Najaf, Iraq; 2 Al-Hakeem Hospital, Al-Najaf Al-Ashraf, Iraq

**Keywords:** Coenzyme Q10, CI/RI, IL-6, IL-10, TNF-α, ICAM-1, NF-κB p65, total anti-oxidant capacity

## Abstract

This study aimed to investigate the potential neuroprotective effects of coenzyme Q10 in cerebral ischemia-reperfusion injury-induced neuronal damage and explore the underlying mechanisms. Twenty-eight adult male rats, weighing approximately 200-300 grams, were randomly divided into four groups: the sham group (neck dissection without ischemia), the control group (30 minutes of bilateral common carotid artery ligation followed by one hour of reperfusion), the vehicle group (oral carboxymethylcellulose solution for seven days prior to bilateral common carotid artery ligation and reperfusion), and the treatment group (seven days of coenzyme Q10 pretreatment followed by bilateral common carotid artery occlusion and reperfusion). Histopathological analysis and measurement of brain infarct size were performed, and cerebral levels of IL-6, IL-10, TNF-α, ICAM-1, NF-κB p65, and total antioxidant capacity were assessed. These cerebral tissue levels and cerebral infarct size were significantly elevated in the control and vehicle groups compared to the sham group. Conversely, the total antioxidant capacity was significantly reduced in these groups. Coenzyme Q10 treatment resulted in a significant increase in IL-10 and total antioxidant capacity levels, along with a significant decrease in IL-6, ICAM-1, TNF-α, and NF-κB p65 levels. Histopathological analysis revealed a significant reduction in ischemic damage in the coenzyme Q10-treated group. Coenzyme Q10 has neuroprotective properties in rats subjected to cerebral ischemia/reperfusion injury, possibly through its anti-inflammatory and anti-oxidative effects.

## INTRODUCTION

Ischemic stroke is a leading cause of death and disability worldwide, contributing to 11.6% of all deaths and 5.7% of total disability worldwide [1]. The oxygen requirements of the nervous tissue are excessively high, making the brain highly susceptible to ischemia. The subsequent reperfusion following cerebral ischemia can precipitate more neurological damage. Cerebral ischemia-reperfusion injury (CI/RI) refers to the reduction of cerebral blood supply followed by the restoration of perfusion. The restoration of cerebral blood flow after ischemia may reverse damaged tissue, but inversely, it can cause more injury and necrosis (reperfusion injury) [1], which presents as infarct progression, neurological deterioration, brain edema, and hemorrhagic transformation of ischemia [2]. This perfusion injury has multifactorial aetiologies. Leukocyte infiltration, calcium overload, oxidative stress, excitotoxicity, mitochondrial damage, platelet and complement activation, and blood-brain barrier disruption are all part of this injury mechanism [3]. The altered blood rheology of slow blood flow and altered shear stress on the endothelial cells after vascular obstruction induces a strong inflammatory cascade [4]. Following an ischemic insult, reactive oxygen species (ROS), cytokines, and chemokines generated by the injured tissue cause leukocyte recruitment and increased expression of adhesion molecules on the brain's endothelium [5]. Neutrophils promote ischemic damage by releasing proteases, reactive oxygen/nitrogen species, and proinflammatory IL-1 [6]. The first opening of the blood-brain barrier occurs within hours following cerebral ischemia due to increased transcytosis. The second opening ensues 24-48 hours later and is attributed to proteolyticLysis of the basement membranes and the tight junctions with loss of endothelial cells. During this later stage, marked peripheral immune cell infiltration is noted [6]. The blood-brain barrier destruction is more frequent in patients with reperfusion (45%) than in brain ischemia without reperfusion (18%) [3]. An imbalance between the antioxidant and oxidative systems results in the process of oxidative stress brought on by either excessive synthesis or inadequate scavenging of the oxygen/nitrogen reactive species [7]. The production of free radicals in CI/RI is continuous along the ischemic stage, while its release during the reperfusion stage is limited to the early phase following oxygen delivery to the ischemic region [8]. Although the brain comprises just 2% of the body's total body weight, it expends 20% of its oxygen production. Therefore, the brain produces more free radicals than any organ. The goal of treating ischemic stroke is to salvage as much of the ischemic penumbra as possible at the earliest opportunity. According to reports, magnetic resonance imaging (MRI) scans have shown that 50% of all patients with acute ischemic stroke still have penumbras [9]. Despite over 700 drugs and extracts explored in preclinical studies, no treatment for post-ischemic cerebral injury has been accepted. Coenzyme Q10 (CoQ10) is a potent antioxidant and free radical scavenger with anti-inflammatory effects [2]. Coenzyme Q10 exists in two forms, oxidized (ubiquinone) and reduced (ubiquinol), and serves as an essential cofactor in mitochondrial electron transport and oxidative phosphorylation, playing a critical role in adenosine triphosphate (ATP) production [10]. This study aimed to investigate the potential neuroprotective effects of coenzyme Q10 in cerebral ischemia/reperfusion injury-induced neuronal damage and explore the underlying mechanisms.

## MATERIAL AND METHODS

The study was conducted at the Department of Pharmacology, College of Medicine, Kufa University. Twenty-eight adult male rats weighing between 200 and 300 grams were housed in the animal facility at the university, with conditions maintained at a relative humidity of 60-65% and a twelve-hour light/dark cycle. Rats were randomly divided into four groups, each consisting of seven rats: sham (surgical procedure without Bilateral Common Carotid Arterial occlusion (BBCAO), control (BCCAO for 30 minutes + one-hour reperfusion), vehicle (seven-day 1% carboxymethylcellulose treatment + BCCAO for 30 minutes + one-hour reperfusion), and treatment (seven-day 40mg/kg CoQ10 pretreatment + BCCAO for 30 minutes + one-hour reperfusion).

### Cerebral ischemia procedure

Cerebral ischemia was induced using the BCCAO method following the administration of general anesthesia. General anesthesia was achieved through an intraperitoneal injection of ketamine and xylazine at 100 mg/kg and 10 mg/kg, respectively. A median neck incision was performed, and both common carotid arteries were exposed and occluded for 30 minutes to induce global cerebral ischemia. Following the 30-minute ischemic phase, the occluding clamps were released, and reperfusion was kept for one hour.

### Preparation of samples

In the final phase of the experiment, the animals were euthanized, and their brains were carefully extracted by skull dissection. The isolated brains were promptly placed on ice to preserve their structural integrity. Subsequently, the brains were coronally sectioned to facilitate various analytical procedures, including enzyme-linked immunosorbent assay (ELISA), histopathological examination, immunohistochemistry (IHC), and staining with Triphenyltetrazolium chloride (TTC) dye. To prepare the brain tissue for analysis, homogenization was performed by combining the brain samples with a mixture of ice and a 0.1 M phosphate buffer solution (PBS) in a ratio of 1:10 (weight/volume). The mixture also contained a protease inhibitor cocktail and 0.2% Triton X-100 [11]. The mixture was centrifuged at 14000xg for 20 minutes at 4^o^C. Then, levels of interleukin-6 (IL-6), IL-10, intercellular adhesion molecule-1 (ICAM-1), tumor necrosis factor-alpha (TNF-α), and total antioxidant capacity (TAC) were assessed using ELISA kits protocols.

### Histopathological examination

Brains were fixed in 10% formalin, sliced by an automated tissue processor, and stained with hematoxylin and eosin. The double-blind approach was used, and the scoring was as follows [12]: 0 (normal): no damage. 1 (mild): slight interstitial edema or dark Eosinophilic neurons. 2 (moderate): at least two small hemorrhages. 3 (severe): local necrosis. Cerebral infarct volume was determined using the immersion method with Triphenyltetrazolium chloride (TTC) stain. The levels of nuclear factor-kappa B (NF-κB) in the cerebral tissue were analyzed using the Dako En Vision immunohistochemistry (IHC) technique.

### CoQ10

The coenzyme Q10 (CoQ10) doses, obtained from Med Chem Express/USA, were prepared immediately. They were dissolved in a 1% concentration of carboxymethylcellulose, and the dosage was calculated at 40 mg/kg/day for administration through oral gavage.

### Statistical analysis

Data was analyzed using SPSS version 26. The Shapiro-Wilk and ANOVA tests were used to determine the normal distribution and analyze data, followed by a post-hoc test. Kruskal-Wallis and post hoc tests were used to evaluate non-parametric data. p-values of 0.05 were considered statistically significant.

## RESULTS

**Figure 1:**
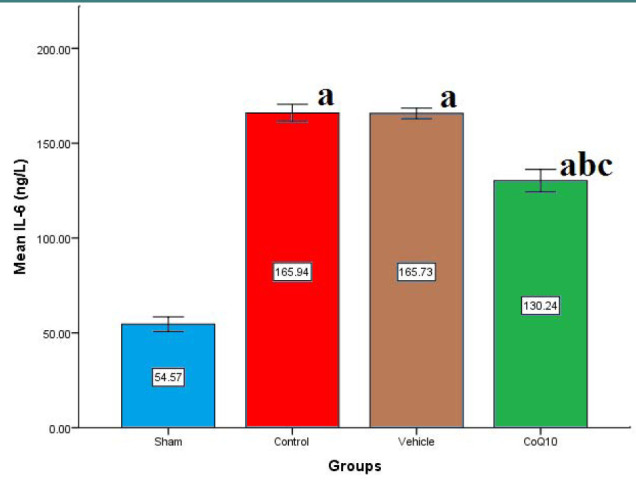
Cerebral IL-6 (ng/L) across groups Mean±SEM was significantly lower in the CoQ10 treated group (p<0.05) compared to the control group.

**Figure 2:**
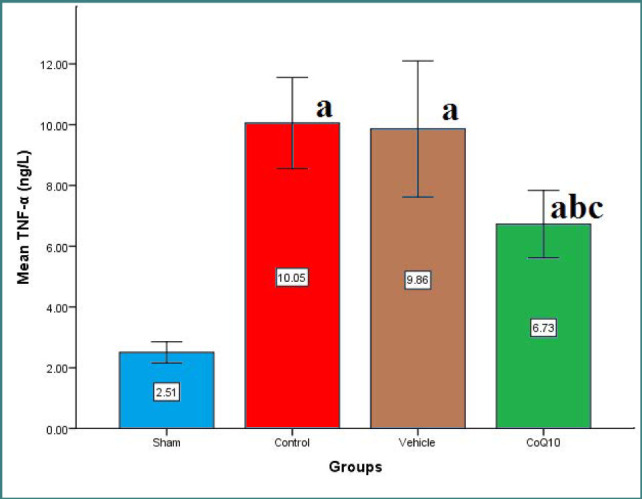
Cerebral TNF-α (ng/L) across groups Mean±SEM was significantly lower in the CoQ10 treated group (p<0.05) compared to the control group.

**Figure 3:**
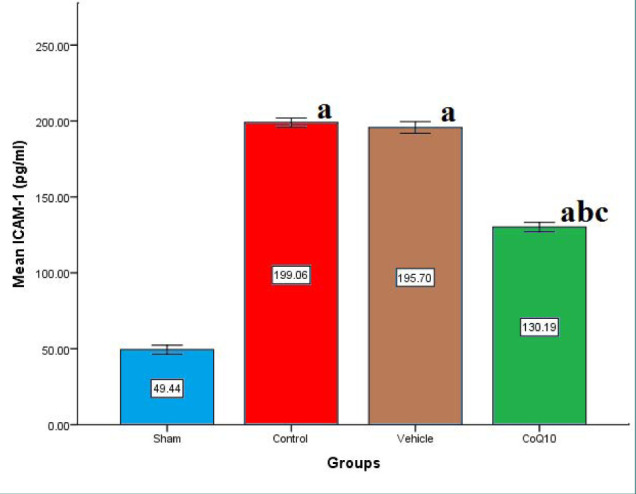
Cerebral ICAM-1 (pg/ml) across groups Mean±SEM was significantly lower in the CoQ10 treated group (p<0.05) compared to the control group.

**Figure 4:**
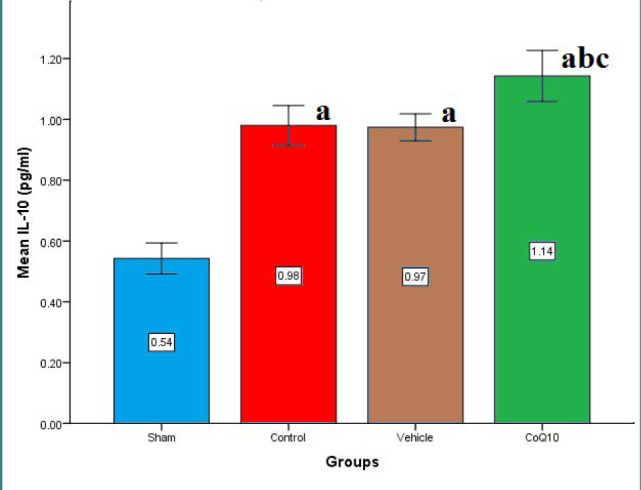
Cerebral IL-10 (pg/ml) across groups Mean±SEM was significantly higher in the CoQ10 treated group (p<0.05) compared to the control group.

Cerebral levels of IL-6, IL-10, TNF-α, and ICAM-1 were significantly increased (p<0.05) in the control and vehicle groups compared to the sham group. In contrast, CoQ10 treatment led to a significant reduction (p<0.05) in the levels of IL-6, ICAM-1, and TNF-α, while IL-10 levels were significantly increased (p<0.05) when compared to the control and vehicle groups (Figures 1-4).

**Figure 5:**
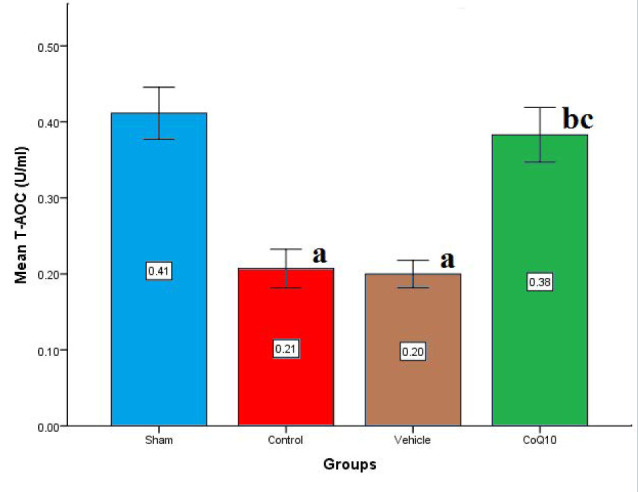
Cerebral TAC (U/ml) across groups Mean±SEM was significantly lower in the CoQ10 treated group (p<0.05) compared to the control group.

The cerebral level of total antioxidant capacity (TAC) was significantly decreased (p<0.05) in the control and vehicle groups compared to the sham group. However, TAC level was significantly increased (p<0.05) in the CoQ10-pretreated compared to control and vehicle groups (Figure 5).

### Effects of CoQ10 treatment on cerebral NF-κB p65 (IHC)

**Figure 6:**
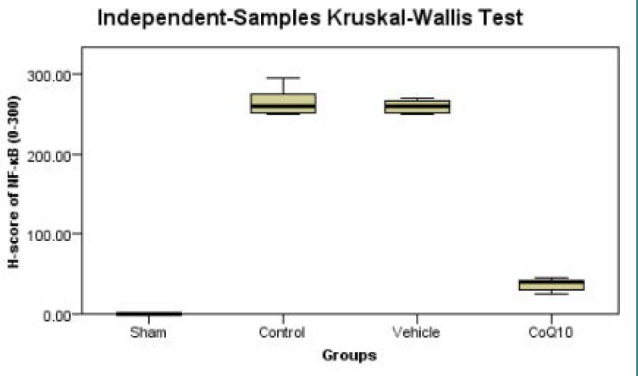
Kruskal Wallis box plot illustrating medians of cerebral NF-κB nuclear expression in the study groups Significantly lower (p<0.05) levels were observed in the CoQ10-treated group compared to the control and vehicle groups.

**Figure 7:**
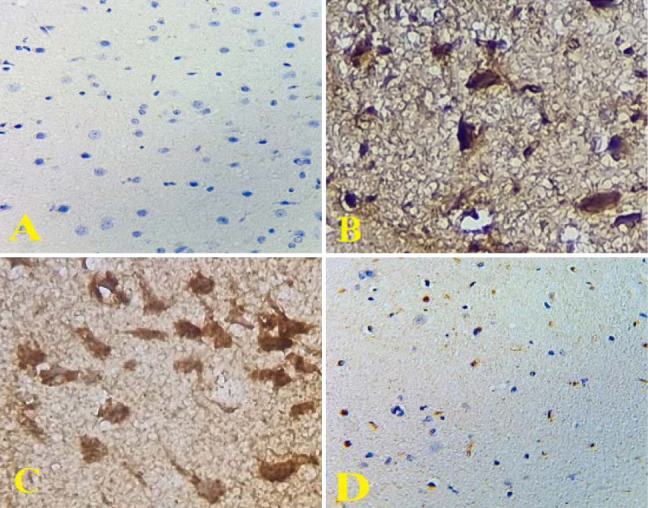
IHC results of NF-κB nuclear expression in rat brain. (A): Sham group: negative expression (X200). (B): Control group and (C): Vehicle group: intense nuclear expression (X400). (D): CoQ10-treated group: weak expression(X400)

In this research, NF-κB p65 nuclear expression was significantly increased (p<0.05) in the control and vehicle groups compared to the sham group. Conversely, CoQ10 treatment resulted in a significant decrease (p<0.05) in NF-κB p65 nuclear expression (Figures 6-7A-D).

### Histopathological results

**Figure 8:**
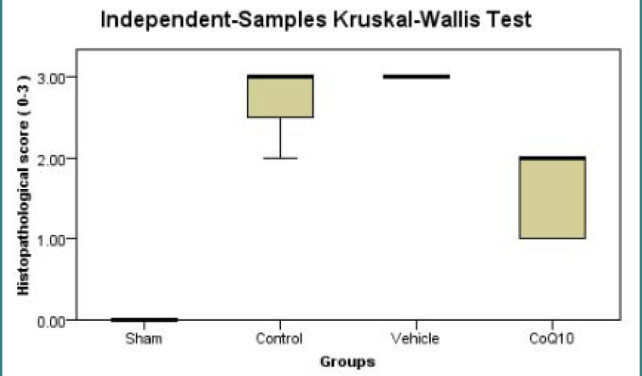
Kruskal Wallis box plot shows medians of ischemic changes in histopathological score in all study groups The coQ10-treated group had a significantly lower score (p<0.05) than the control group.

**Figure 9:**
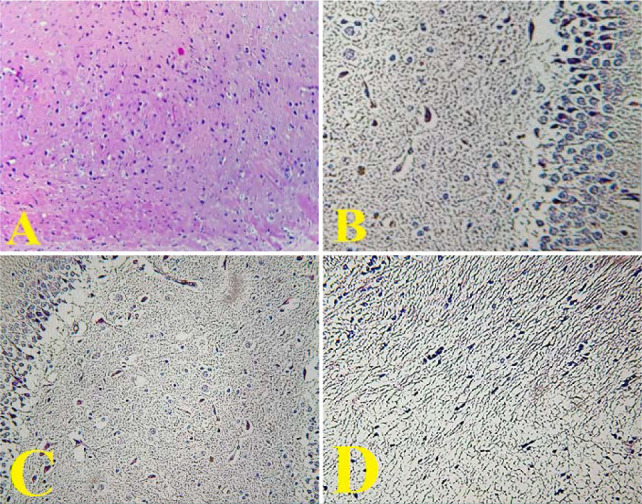
Cross section of rat brain stained with hematoxylin and eosin. (A) Sham group: normal histology of rat brain (100X). (B) Control group and (C) Vehicle group: areas of necrosis (100X). (D) CoQ10-treated group: interstitial edema (100X)

The control group had significantly severe (p<0.05) ischemic injury, while the CoQ10-treated group demonstrated a substantial reduction (p<0.05) in the histopathological scoring of ischemic injury (Figures 8-9 A-D).

### Cerebral infarct size results

**Figure 10:**
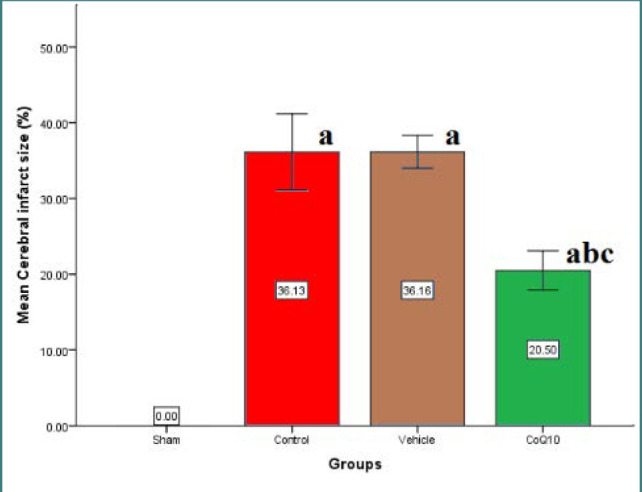
Score of cerebral infarct size (%) Mean±SEM was significantly lower in the CoQ10-treated group (p<0.05) compared to the (b) control group.

**Figure 11:**
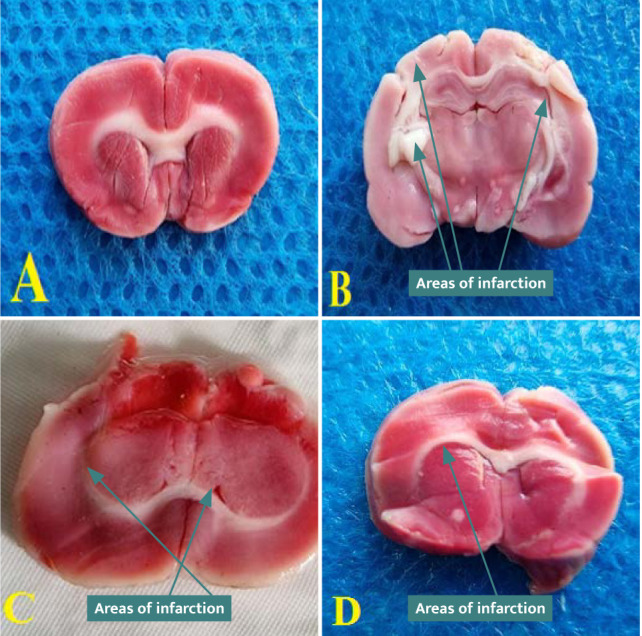
Cerebral coronal sections stained with TTC stain. (A) Sham group. (B) Control group. (C) Vehicle group. (D) Treatment group.

There was a significant increase (p<0.05) in the cerebral infarction score size in the control and vehicle groups compared to the sham group. Cerebral infarction size score was significantly reduced (p<0.05) in the CoQ10-pretreated group (Figures 10 – 11A-D).

### Discussion

Inflammation is a main characteristic of stroke pathogenesis [13]. TNF and IL-6 are potent, proinflammatory cytokines that significantly increase within the initial 24 hours following a stroke [14]. Moreover, experimental stroke models in rodents have shown that these cytokines influence the extent of ischemic injury, making them promising therapeutic targets for stroke therapy [15]. Oxidative stress represents a major mechanism underlying neuronal damage induced by cerebral ischemia/reperfusion injury (CI/RI) [16].

### Effects of CoQ10 on cerebral inflammation and antioxidant status

In our study, CoQ10 treatment led to significant reductions (p<0.05) in cerebral levels of IL-6, ICAM-1, TNF-α, and NF-κB p65, while significantly increasing TAC and IL-10 levels (p<0.05) compared to the control and vehicle groups. This aligns with the findings of Abdl El-Aal *et al*. [17], who observed similar reductions in hippocampal TNF-α, ICAM-1 levels, and NF-κB p65 expression in the CoQ10-pretreated rats subjected to transient BCCAO, compared to a control group. The anti-inflammatory properties of CoQ10 have been demonstrated in hepatic toxicity of acetaminophen [18], testicular injury by sodium arsenate [19], and myocardial toxicity induced by doxorubicin. Cerebral ischemia results in a significant decrease in intrinsic CoQ10 in the brain [20, 21]. Furthermore, it is well known that CoQ10 has strong antioxidant properties and can enhance the antioxidant defense enzyme capacity [22]. CoQ10 reduces neuronal injury by decreasing the production of free radicals and inhibiting lipid peroxidation, a main cause of injury induced by free oxygen radicals [23]. To our knowledge, no prior studies have explored the specific effects of CoQ10 on cerebral IL-6, IL-10, and TAC levels in rats exposed to CI/RI.

### Effects of CoQ10 on cerebral histopathology

The CoQ10-treated group showed a significant improvement in ischemic changes. Lu *et al*. [24] showed that ischemic changes were significantly ameliorated in CoQ10-pretreated diabetic rats compared to the control group subjected to total middle cerebral artery occlusion. As far as we know, there are no studies on the CoQ10 effects on cerebral histopathology in healthy rats exposed to CI/RI.

### Effects of CoQ10 on cerebral infarct size

Cerebral infarction size score was significantly reduced (p<0.05) in the CoQ10-pretreated group. Similarly, Obolenskaia *et al*. [21] demonstrated that brain infarction volume, measured by TTC stain, in CoQ10-pretreated rats, was considerably lower than in the acute total middle cerebral artery occlusion control group.

## CONCLUSION

This study identified that CoQ10 can ameliorate cerebral infarct size and ischemia/reperfusion injury. The protective effects of CoQ10 may arise from anti-inflammatory and antioxidant properties.
